# Notes and News

**Published:** 1900-03-17

**Authors:** 


### Notes and News.

### HOSPITAL MEETINGS.

St. Mary's Hospital.—Annual Meeting at half-past four p.m., on Friday, March 16th, at the Hospital.

Hampstead Hospital.—Annual Meeting at four p.m., on Saturday, March 17th, at the Vestry Hall, Haverstock Hill.

Poplar Hospital.—Annual Meeting at four p.m., on Tuesday, March 20th, at the Hospital.

University College Hospital.—Annual Meeting at four p.m., on Wednesday, March 21st, at the Hospital.

Royal London Ophthalmic Hospital.—Annual Meeting at half-past three p.m., on Wednesday, March 21st, at the Hospital (City Road).

Hospital and Home for Incurable Children, 2, Maida Vale — Annual Meeting at three p.m., on Saturday, March 17th, at the Hospital.

Measles are very prevalent amongst the troops at Aldershot, and have interfered in the case of some companies with orders to embark for South Africa.

The Poplar Union have found that vaccination under the new Act is expensive. Last year the fees paid to vaccination officers amounted to £675, whilst in 1898 only £44 3s. 6d. was paid.

The Metropolitan Asylums Board have decided to increase the rate to 2½d. against 2¼d. in the pound. No one can deny that in its fever hospitals the metropolis possesses magnificent and efficient institutions, and this knowledge must console the ratepayer for the enormous expenditure which has been incurred. Besides bringing up the hospital accommodation to its present standard, the Board are now undertaking the maintenance of imbeciles, and providing homes for children.

Lieut.-Colonel Bloomfield Connolly, M.D., in medical charge of the Hospital for Soldiers' Wives at Woolwich, writes to say that on Monday morning an artilleryman's wife gave birth to three male children, and that the mother and the three babies are doing well. The father is now on active service in South Africa, and the mother proposes to name her little "soldiers of the Queen" Victor, Roberts, and Buller respectively, in honour of our Most Gracious Majesty the Queen and her two illustrious generals.

The Manchester Royal Infirmary has now consented to admit female medical students to hospital practice and clinical instruction upon the same terms as the men, the surgical out-patients' department being excepted.

The Board of Health of one of the cities of the United States recently closed a public library during an epidemic of diphtheria and scarlet fever. It was stated that one case of fever had been definitely traced to the contagion of a book from the library.

A new out-patients department has been added to the Stanley Hospital, Liverpool. It contains a waiting-room 75 ft. long by 24 ft. wide, and adjoining are the usual rooms and offices. The cost, partly defrayed by the Roger Lyon Jones' Trust, was £2,630.

When the death-rate was so high a little time ago we pointed out how surely, in a fairly healthy place, a high death-rate is followed by a low one. Last week the death-rate in London was down to 18·8, considerably below the mean for the corresponding period.

In a long letter on the subject of the convalescent homes at Sandgate, Mr. J. J. Jones informs us that these homes are the property of his daughters, the premises being rented by them, and that they are not supported by donations or subscription, but are self-supporting.

Professor Hughes, F.R.S., late President of the Institute of Electrical Engineers, made a will largely in the interests of the Middlesex, the London, King's College, and Charing Cross Hospitals. The will contains some definite conditions. If any one of the four hospitals benefited shall not keep 100 beds continuously open the trustees are to cease distributing to it any of the money from the "David E. Hughes Hospital Trust Fund." Further, he has desired that each hospital, after the expiration of a period of 21 years, shall place one-third of the annual grant from his fund to capital account. It appears likely that the fund will prove to equal £300,000, and later, when no longer subject to the payment of annuities to various legatees, to £400,000.

An interesting lecture was delivered in the Carpenters' Hall, London Wall, on Thursday, 8th inst., by Mr. Wm. Henman, F.R.I.B.A., dealing with the architecture and construction of modern medical hospitals. The lecture was illustrated by lantern photographs, giving views and plans of various institutions all over the country. Beginning with a badly-planned cottage hospital, Mr. Henman showed how it was possible to use the maximum of space while obtaining the minimum of good result, and, by contrasting this with a well-arranged building of a similar character, pointed out how exactly the opposite could be obtained by intelligent adaptation of plans to the requirements of the institution as taught by experience. From these as a nucleus he went on through a series of plans of the most varied size and character, indicating the defects and advantages of each. He expressed his indebtedness to Sir Henry Burdett's "Hospitals," and to the treatise by the late Sir Douglas Galton, entitled "Healthy Hospitals."

